# Mutations that confer resistance to sodium azide affect the levels of two physiological isoforms of SecA in Escherichia coli

**DOI:** 10.1099/mic.0.001707

**Published:** 2026-05-15

**Authors:** Chen Jiang, Mathew Milner, Georgia Williams, Osama Abdullah, Peter Lund, Damon Huber

**Affiliations:** 1 School of Biosciences, University of Birmingham, Edgbaston, Birmingham, UK

**Keywords:** azide, C-terminal tail, protein translocation, Sec, SecA, TraDIS

## Abstract

Sodium azide inhibits bacterial growth by inhibiting the SecA-dependent translocation of proteins across the cytoplasmic membrane, and all of the mutations known to confer increased resistance to azide are in the *secA* gene. However, the molecular mechanism of resistance is unclear. To gain insight into this mechanism, we designed a genetic screen to isolate transposon insertion mutations that conferred increased resistance to sodium azide, which were also unlinked to *secA*. We isolated six mutants, which we dubbed *uar* mutants, containing insertions in the *rpsA*, *rpsQ*, *srmB* and *nusB* genes. Growth of *Escherichia coli* in the presence of azide causes increased accumulation of the larger of two previously noted, but uncharacterized, isoforms of SecA. However, the *uar* mutations caused increased production of the smaller isoform in the presence of azide. Mutations in the *secA* gene that confer azide resistance also caused increased production of the small SecA isoform in the presence of azide, suggesting a shared mechanism. Expression of truncated variants of SecA, which lack the C-terminal tail, caused moderately increased resistance to azide, and Western blotting analysis confirmed that the smaller SecA isoform lacks the C-terminal ~70 amino acids. These results indicate that *E. coli* produces two SecA isoforms under normal growth conditions and that changes in the proportions of the isoform correlate with resistance to sodium azide.

## Introduction

The ATPase SecA is an essential component of the bacterial Sec machinery, which is responsible for transporting a subset of newly synthesized proteins across the cytoplasmic membrane [1,3]. Rounds of ATP binding and hydrolysis by SecA drive translocation of these proteins through a channel in the cytoplasmic membrane formed by the integral membrane proteins SecY, SecE and SecG [4,5]. The N-terminal ~830 amino acids of SecA from *Escherichia coli* form the evolutionarily conserved catalytic core of the protein, which (i) binds to substrate polypeptides, (ii) binds to SecYEG, (iii) contains the ATPase activity and (iv) is sufficient to drive translocation through SecYEG [5]. The SecA proteins of most bacterial species additionally contain a relatively long C-terminal tail (CTT) consisting of two domains: a structurally flexible linker domain (FLD; amino acids ~832–880 in *E. coli*) and a small metal-binding domain (MBD; amino acids ~880–901) [6]. The MBD facilitates the interaction of SecA with SecB (a molecular chaperone that assists Sec-dependent translocation in Gram-negative bacteria) and with ribosomes [6,7], and its structure is stabilized by binding to a metal ion (ferrous iron or zinc) [8,9]. The FLD tethers the MBD to the C-terminus of the SecA catalytic core and influences the structure and activity of the catalytic core by binding in the same site where SecA binds to substrate polypeptides [6,10].

Sodium azide inhibits bacterial growth by inhibiting SecA-dependent protein translocation [11,13]. Mutations that confer resistance to azide occur almost exclusively in the *secA* gene [14,17]. Because most of these mutations alter amino acids in the two nucleotide-binding domains of SecA, it has been suggested that azide inhibits the ATPase activity of SecA at the step of nucleotide exchange, analogous to the mechanism of action of azide on the F-ATPase [14,15, 18, 19]. Indeed, many of the mutations that confer azide resistance also increase the rate of nucleotide exchange [20]. Nonetheless, a definitive mechanism of action of azide *in vivo* has not been established. For example, in addition to SecA and the F-ATPase, azide also inhibits several other enzymes, including cytochrome *bo* oxidase [21,22] and catalase [23], and mutations at multiple other unidentified loci can cause azide resistance in *Proteus hauseri* [24]. Furthermore, the concentration of azide required to inhibit SecA in biochemical assays is at least tenfold higher than that required to inhibit SecA-dependent translocation *in vivo* [25], suggesting that growth inhibition might not be caused exclusively by inhibition of SecA.

To gain insight into the effect of azide on the bacterial cell, we screened for mutations in *E. coli* that conferred azide resistance, which were unlinked to the *secA* gene. We isolated six transposon insertion mutations that conferred increased resistance to sodium azide, all of which affected genes involved in ribosome biogenesis. Our results led us to the finding that subinhibitory concentrations of azide caused the accumulation of the larger of the two previously noted SecA isoforms [26]. All of the azide-resistant mutants that we investigated, including those that contained mutations in the *secA* gene, caused increased levels of the smaller isoform relative to the larger isoform in the presence of azide. Subsequent analysis revealed that the larger isoform corresponded to full-length SecA, while the smaller isoform lacked the CTT. These results suggest that *E. coli* produces both isoforms of SecA under physiological growth conditions and that changes in their abundance are correlated with differences in resistance to azide.

## Methods

### Chemicals and growth media

All chemicals were purchased from Sigma-Aldrich (Merck) unless otherwise indicated. Rabbit anti-SecA antiserum was a lab stock. Rabbit anti-MBP antibody was obtained from New England Biolabs (Ipswich, Massachusetts). HRP-coupled anti-rabbit-IgG was purchased from Sigma-Aldrich (Merck). HRP-coupled streptactin was purchased from IBA Lifesciences (Göttingen, Germany). Unless otherwise indicated, bacteria were grown using lysogeny broth (LB) media [27]. For recovery after electroporation, cells were grown using super optimal broth with catabolite repression medium (SOC). To select for leucine prototrophy, cells were grown on M63 minimal plates containing glucose as a carbon source [27]. Top agar was prepared using 7 g l^−1^ agar, 10 g l^−1^ tryptone and 8 g l^−1^ NaCl. Where indicated, media were supplemented with 200 µg ml^−1^ ampicillin or 50 µg ml^−1^ kanamycin after autoclaving. Sodium azide was added at the indicated concentration after autoclaving.

### Strain and plasmid construction

*E. coli* strains and plasmids used in this study are listed in Table 1. Strain MG1115 was a kind gift from M. Grabowicz and T. Silhavy. Strain DRH1292 was constructed by transforming DRH955 with plasmid pCP20 and streaking at 42 °C to induce expression of the FLP recombinase [28]. Chromosomal mutations were transduced using P1 as previously described [27]. Gibson Assembly (New England Biolabs, Ipswich, Massachusetts) was used to construct the *secA-yfp* gene fusion in pLH1 by fusing the initiation codon of the *yfp* open reading frame to the final sense codon of the *secA* open reading frame on plasmid pDH692. To construct strain DRH991, the *secA-yfp* gene from plasmid pLH1 was integrated onto the chromosome of strain DRH663 using λInCh, and the *secA* containing plasmid was cured by selecting for resistance to 1 mM Isopropyl-β-D-thiogalactoside (IPTG) (IPTG-induced over-expression of *secA* from the plasmid is toxic).

**Table 1.** Strains and plasmids used in this study

Strain	Description	References/source
MG1655	*E. coli* K-12 F^-^ λ^-^ *rph-1*	Lab stock
MC4100	F- λ^-^*araD139* Δ(*argF-lac)169 flhD5301* Δ(*fruK-yeiR)725 relA1 rpsL150*^strR^ *rbsR22* Δ(*fimB-fimE)632 deoC1*	[55]
DHB4	F′ *lac-pro lacl*^q^*/ araD139* Δ(*ara-leu*)*7697 galU galK ΔlacX74 rpsL thi* Δ*malF3* Δ(*phoA*[*PvuII*]) *phoR*	[56]
BW25113	F- Δ(*araD-araB)567 rrnB3* Δ*lacZ4787 rph-1* Δ(*rhaBAD)568 hsdR514*	[28]
MG1115	MC4100 Ara+ *secA827*::IS*1.leuA*::Tn*10*	[36]
JW0073	BW25113 Δ*leuA*::*aphA*	[57]
SS3242 (CGSC6909)	F-, *secA219*(aziR), *pro-48*, *lacZ118*(Oc), *lacI22*, *λ^-^*, *trpA9605*(Am), *his-85*(Am), *gyrA19*(NalR), *rpsL171*(strR), *metE90*, *zjj-2002::Tn10*, *trpR55*	*E. coli* Genetic Stock Center (CGSC; Yale University, New Haven, Connecticut)
DRH745	MC4100 Δ*secA* λ-p_Trc_-s*ecA^+^*	This study
DRH839	MC4100 Δ*secA* λ-p_Trc_-s*ecA-biotin* Spec^R^	[37]
DRH856	MG1655 Δ*tig*::*cat*	This study
DRH952	MG1655 Δ*dsbC*::*aphA*	This study
DRH955	MG1655 Δ*dsbC*::*aphA* Δ*tig*::*cat*	This study
DRH991	MC4100 Δ*secA*::*aphA* λ*att*::pTrc(DSW204)-*secA-yfp*	This study
DRH997	MC4100 *secA-D1*(Azi^R^) (C108667T resulting in amino acid substitution T130I)*	This study
DRH998	MC4100 *secA-D2*(Azi^R^) (T108834G resulting in amino acid substitution Y186D)*	This study
DRH1014	MC4100 Δ*leuA*::*aphA*	This study
DRH1070	MC4100 λ-p_Trc_-*secA*Δ*CTT*	[6]
DRH1075	MC4100 *secA827*::IS*1.leuA*::Tn*10*	This study
DRH1116	BW25113 *secA-D1*	This study
DRH1122	BW25113 *secA219* (C109821T; L515F)*	This study
DRH1127	BW25113 *secA-D2*	This study
DRH1292	MG1655 Δ*tig* Δ*dsbC* (Kan^S^ Cm^S^)	This study
CJ101	DRH1292 *uar-1* (*rpsQ*::miniTn5)	This study
CJ102	DRH1292 *uar-2* (*rpsQ*::miniTn5)	This study
CJ103	DRH1292 *uar-3* (*rpsA*::miniTn5)	This study
CJ104	DRH1292 *uar-4* (*rpsQ*::miniTn5)	This study
CJ105	DRH1292 *uar-5* (*srmB*::miniTn5)	This study
CJ106	DRH1292 *uar-6* (*nusB*::miniTn5)	This study
CJ107	MG1655 *uar-1* (*rpsQ*::miniTn5)	This study
CJ108	MG1655 *uar-2* (*rpsQ*::miniTn5)	This study
CJ109	MG1655 *uar-3* (*rpsA*::miniTn5)	This study
CJ110	MG1655 *uar-4* (*rpsQ*::miniTn5)	This study
CJ111	MG1655 *uar-5* (*srmB*::miniTn5)	This study
CJ112	MG1655 *uar-6* (*nusB*::miniTn5)	This study
CJ113	BW25113 *uar-1* (*rpsQ*::miniTn5)	This study
CJ114	BW25113 *uar-2* (*rpsQ*::miniTn5)	This study
CJ115	BW25113 *uar-3* (*rpsA*::miniTn5)	This study
CJ116	BW25113 *uar-4* (*rpsQ*::miniTn5)	This study
CJ117	BW25113 *uar-5* (*srmB*::miniTn5)	This study
CJ118	BW25113 *uar-6* (*nusB*::miniTn5)	This study
**Plasmid**	**Description**	**Reference**
pTrc99a	Medium copy number plasmid containing p*_trc_* promoter for IPTG-inducible protein expression and ampicillin resistance marker	Invitrogen
pDSW204	Derivative of pTrc99a containing *lac*UV5 promoter	[58]
pCP20	Plasmid expressing FLP recombinase under temperature-dependent promoter and containing temperature-sensitive origin of replication. Ampicillin and chloramphenicol resistance markers	[28]
pHK771	pTrc99a containing IPTG-inducible *phoA* gene	[59]
pDH736	Derivative of pHK771 expressing full-length SecM fused to PhoA	[37]
pLH1	pTrc99a expressing SecA-YFP fusion protein	This study

* Position of base substitution in azide-resistant *secA* mutants is given according to position in reference sequence NC_000913.3. The position of the resulting amino acid substitution is given according to the methionine start codon.

### Azide susceptibility assays

Susceptibility to azide was determined by the filter disc assay. An overnight culture of the indicated bacterial strain was spread evenly onto an LB agar plate using a cotton swab to create an even lawn. Then, a 6 mm antibiotic assay filter disc (Merck Millipore) was placed in the centre of the lawn, and 10 µl of 0.5 M sodium azide was pipetted onto the filter disc, and the sensitivity was determined by comparing the average diameters of the zones of clearing caused by the antimicrobial compound. Sensitivity to sodium azide was also determined by spreading cultures of *E. coli* BW25113 onto plates containing 0.25, 0.5, 0.75, 1.0 and 1.5 mM sodium azide to determine the minimum concentration that prohibited growth of individual colonies or by growing *E. coli* in culture at 37 °C in the presence of the indicated concentration of sodium azide. Growth was monitored by measuring the optical density at 600 nm every 60 min for 15 h.

### Transposon mutagenesis

Transposon mutant libraries were constructed by transforming strains MG1655, DRH856, DRH952 and DRH1292 with a mini-Tn*5* transposon. Cells were made electrocompetent by washing twice with sterile deionized water and twice with 10% glycerol and resuspending in 1/1,000 vol. of 10% glycerol. Thirty microlitres of the competent cells were then transformed with 0.1 µl EZ-Tn5 <KAN-2> transposome kit (Cambio, Cambridge, UK) in a 1 mM gap cuvette using an Eppendorf Eporator using 1.7 kV and recovered for 60 min at 37 °C in SOC media. Cells containing stable insertions were selected on plates containing kanamycin. The resulting transformants were pooled by flooding the plates with 10% glycerol and stored at −80 °C. To ensure sufficient coverage of the *E. coli* chromosome, greater than 500,000 independent transformants were isolated for each strain.

### Genetic screen for azide-resistant transposon mutants

To select for azide-resistant mutants, tenfold serial dilutions of the transposon mutant library of DRH1292 were grown on LB plates containing 0.5, 0.75, 1.0 and 1.5 mM sodium azide or lacking sodium azide. Single colonies were selected from azide-containing plates that produced a lawn on plates lacking azide at the same dilution of cells and restreaked onto plates containing the same concentration of azide. To confirm that azide resistance was stable, the cells were grown in the absence of selection on LB plates lacking azide and then restreaked onto plates containing azide. Plates containing at least 1 mM azide produced azide-resistant colonies with a stable phenotype. To confirm that the mutation was linked to the transposon insertion, bacteriophage P1 was grown on the mutants, and the lysate was used to transduce the indicated bacterial strains by selecting for kanamycin resistance.

### Genetic screen for azide-resistant *secA* mutants

Novel azide-resistant mutations were isolated as spontaneous mutants as described previously [14]. Briefly, overnight cultures were spread on plates containing 3 mM sodium azide. Individual colonies that appeared after 2–3 days of incubation at 37 °C were restreaked onto LB with and without 2 mM sodium azide to confirm azide resistance. Azide resistance genes were transduced into MC4100 and BW25113 by transducing strains JW0073 and DRH104 and selecting for leucine prototrophy on M63 minimal plates and screening for azide resistance and kanamycin sensitivity. The point mutations that conferred azide resistance were identified by whole-genome sequencing (MicrobesNG, Birmingham, UK).

### Identification of transposon insertion sites in azide-resistant mutants

For most of the azide-resistant transposon mutants, it was possible to amplify the DNA adjacent to the mini-Tn*5* <Kan> insertion sites by arbitrary PCR as previously described [29]. A colony of the indicated mutant was resuspended in 100 µl nuclease-free water to extract the genomic DNA. The reaction mixtures contained 2X MyTaq Red mastermix (Bioline), 5 µl of the extracted genomic DNA, nuclease-free water, 1 µM HIB17 (CGGAATTCCGGATNGAYKSNGGNTC) and 1 µM KAN-2_FOR (ACCTACAACAAAGCTCTCATCAACC) or KAN-2_REV (GCAATGTAACATCAGAGATTTTGAG). The DNA fragments were then amplified using touchdown PCR using an initial annealing temperature of 60 °C and decreasing the annealing temperature by 1 °C each cycle for 25 cycles. The seeded PCR reaction was then subjected to a further 25 cycles using an annealing temperature of 45 °C. PCR reactions that produced products were analysed using Sanger sequencing (Source BioScience, Nottingham, UK) using the KAN-2_FOR or KAN-2_REV primer to determine the site of the insertion. The position of the insertion of the *uar-5* mutation was determined by whole genome sequencing (MicrobesNG, Birmingham, UK). The locations of all the insertion sites were confirmed by PCR using primers flanking the insertion site.

### Transposon-directed insertion-site sequencing

The locations of the transposon insertions in each of the pooled libraries were determined using transposon-directed insertion-site sequencing (TraDIS) as previously described [30]. Genomic DNA was extracted using a STRATEC SE (Birkenfeld, Germany) RTP Bacteria DNA Mini kit using ~10^9^ cells. The extracted DNA (2 µg µl^−1^ in nuclease-free water) was sheared using a Diagenode Bioruptor sonicator using 30 s pulses at low intensity with 90 s rests for 13 cycles. The sheared samples were then concentrated to 55.5 µl using an Eppendorf vacuum concentrator. The sheared DNA was then prepared for Illumina sequencing using a NEBNext Ultra DNA Library Prep Kit (New England Biolabs, Ipswich, Massachusetts). Solid-phase reversible immobilization (SPRI) beads were prepared as previously described [30,31] and used to select for DNA fragments with a size of ~250 kb. The extracted DNA was used as a template to amplify transposon-adjacent DNA in a PCR reaction using NEBNext Q5 Hot Start HiFi PCR Master Mix and primers TKK_F (ACCTGCAGGCATGCAAGCTTCAGG) and TKK_R (GACTGGAGTTCAGACGTGTGCTCTTCCGATC). The PCR product was purified using SPRI beads and amplified a second time using NEBNext Q5 Hot Start HiFi PCR Master Mix to add a custom inline barcode [30] and Illumina-specific homology (NEBNext Multiplex Oligos for Illumina, New England Biolabs). The prepared DNA was sequenced using an Illumina platform by Novogene UK (Cambridge, UK). The DNA sequencing data were then analysed using previously described custom scripts and those included in the BioTraDIS package [30,32]. The genomic sequence for *E. coli* MG1655 (NCBI accession NC_000913.3) was used as a reference sequence for matching sequencing reads. Raw fastq files of TraDIS were deposited in the European Nucleotide Archive under the study accession number PRJEB103834.

### PhoA assays

PhoA activities were determined as previously described [33]. Cells containing plasmid pDH736 were grown in LB containing ampicillin, 0.5 mM IPTG and 0.5 mM sodium azide at 37 °C to OD_600_ ~0.5 and transferred to ice for 20 min to stop growth. The cells were centrifuged and resuspended in 100 mM Tris-HCl, pH=8 and permeabilized by the addition of 2 drops of chloroform and 1 drop of 0.1% SDS. The permeabilized cells were then incubated at 37 °C for 5 min to equilibrate the temperature. A total of 0.2 ml PNPP (4 mg ml^−1^) was added to each lysate and mixed by inversion until a yellow colour could be detected by eye. The reaction was then stopped by the addition of 0.5 ml 1M K_2_PO_4_ and cooling on ice. Samples were centrifuged for 1 min at 13,000 r.p.m. to remove the cell debris, and the absorbance of each sample was determined at 550 and 420 nm. The PhoA activity was calculated using the equation:


$$\text{Unit of PhoA activity}\;(MU)=\frac{1000x\;\left(Abs_{420}-1.75\;x\;Abs_{550}\right)}{t\;\;x\;\;v\;x\;Abs_{600}}$$

Where t=reaction time (minutes), v=volume of culture (ml) and Abs_420_, Abs_550_ and Abs_600_ were the absorbance readings at 420, 550 and 600 nm, respectively.

### SDS-PAGE and Western blotting

Steady-state concentrations of SecA were determined by SDS-PAGE and Western blotting, as previously described. Cells grown in culture were resuspended in 1X Laemmli buffer [34] to an equivalent concentration of OD_600_=10 and lysed by incubation at 100 °C for 5 min. Equal volumes of each sample were resolved using SDS-PAGE with a 12% gel and then transferred to a nitrocellulose membrane. The membranes were then blocked using 5% milk casein and probed using rabbit anti-SecA primary antiserum and HRP-coupled donkey anti-rabbit secondary antiserum. The resulting bands were visualized on a BioRad GelDoc XR+ gel imager using a high-sensitivity ECL kit (Cytiva).

## Results

### Generation of transposon mutant library

To gain insight into the mechanism of action of azide *in vivo*, we sought to isolate mutations that conferred resistance to azide that were unlinked to the *secA* gene. To avoid mutations in *secA*, we considered two possible explanations for the preponderance of *secA* mutations isolated in previous studies.

First, most parental *E. coli* K-12 strains have a relatively high intrinsic resistance to azide, raising the possibility that only mutations in *secA* can confer a sufficient level of resistance required for growth in the presence of the high concentrations of azide used in genetic screens (typically ~3 mM) (e.g. see Huie and Silhavy [14]). That is, while mutations at other loci might confer moderate increases in resistance, these increases might be insufficient to permit growth at such high azide concentrations. However, lower concentrations of azide would make it difficult to distinguish between azide-resistant mutants and background growth. To address this issue, we used a strain of *E. coli* K-12 str. MG1655 containing deletion mutations of the *tig* and *dsbC* genes. Previous work suggested that insertions inactivating *tig* and *dsbC* moderately increase the susceptibility of *E. coli* [9]. Consistent with these findings, a filter disc containing 5 µmol of sodium azide created moderately larger zones of clearing in lawns of the MG1655 containing a Δ*tig* (diameter=3.4 cm) or Δ*dsbC* (3.5 cm) deletion mutation compared to the parent (3.1 cm). Furthermore, combining these mutations in MG1655 Δ*tig* Δ*dsbC* resulted in an even larger zone of clearing (3.8 cm). Both mutations also increased the growth defect caused by the presence of 0.5 mM sodium azide in liquid growth media (Fig. S1, available in the online Supplementary Material). To determine the minimum selective concentration of azide for the double mutant, we grew MG1655 Δ*tig* Δ*dsbC* on LB plates containing 0.25 mM to 2 mM sodium azide. While at least 1 mM azide was required to inhibit growth of the parent strain completely, growth of MG1655 Δ*tig* Δ*dsbC* was completely inhibited by 0.75 mM sodium azide.

Second, we considered the possibility that mutations in genes unlinked to *secA* that cause azide resistance might be rare in comparison to those in *secA*, biasing screens towards mutations in *secA*. To counter this potential bias, we randomly mutagenized the genome of MG1655 Δ*tig* Δ*dsbC* using transposon mutagenesis. To our knowledge, all of the known *secA* mutations that confer azide resistance alter amino acids in the catalytic core of SecA, predominantly in the domains that contain its ATPase activity [14]. However, the region encoding the catalytic core of SecA (i.e. the N-terminal ~827 amino acids) is essential for viability [9], and transposon mutant libraries of *E. coli* do not contain insertions in this region [30]. We therefore generated a library of >500,000 independent mini-Tn*5* transposon mutants of MG1655 Δ*tig* Δ*dsbC*.

### TraDIS analysis of *E. coli* MG1655 Δ*tig* Δ*dsbC* transposon mutant library

To confirm the coverage of transposon mutations in the MG1655 Δ*tig* Δ*dsbC* transposon mutant library, we determined the transposon insertion sites in the library using TraDIS [30]. This analysis detected 341,181 independent insertions in the MG1655 Δ*tig* Δ*dsbC* library, consistent with a transposon insertion every 13–14 bp on average. The large number of mapped insertions also made it possible to determine which genes are essential for viability in these strains with a high degree of confidence (data S1). (Because transposons disrupt gene function, regions of the genome that are essential for viability are devoid of transposon insertions.) An analysis of the insertion profile indicated that MG1655 Δ*tig* Δ*dsbC* contained 451 essential genes (data S1A).

We also generated transposon mutant libraries of the parental strain (MG1655), MG1655 Δ*tig* and MG1655 Δ*dsbC* and analysed the libraries using TraDIS. These results detected 574,250 independent insertions in the parental MG1655 library, 413,069 independent insertions in the MG1655 Δ*tig* library and 28,916 independent insertions in the MG1655 Δ*dsbC* library (Table 2). Analysis of these data indicated that MG1655 contained 336 essential genes, consistent with the number of essential genes in *E. coli* K-12 strain BW25113 (data S1A) [30], and MG1655 Δ*tig* contained 397 essential genes (data S1B). The low number of mapped insertion sites in the MG1655 Δ*dsbC* library made it difficult to predict whether a given gene was essential with a high degree of confidence.

**Table 2.** Unique mini-Tn*5* insertion sites in transposon libraries

Strain	Unique mapped insertion sites*	Predicted essential genes†
MG1655	574250	336
MG1655 Δ*tig*	413069	397
MG1655 Δ*dsbC*	28916	ND‡
MG1655 Δ*tig*Δ*dsbC*	341181	451

* The position of each transposon insertion was determined by TraDIS and alignment to the reference sequence for *E. coli* K-12 strain MG1655 (NC_000913.3).

† Gene essentiality was predicted using BioTraDIS [32] and manual inspection of the insertion sites.

‡ Not determined.

**Fig. 1.**
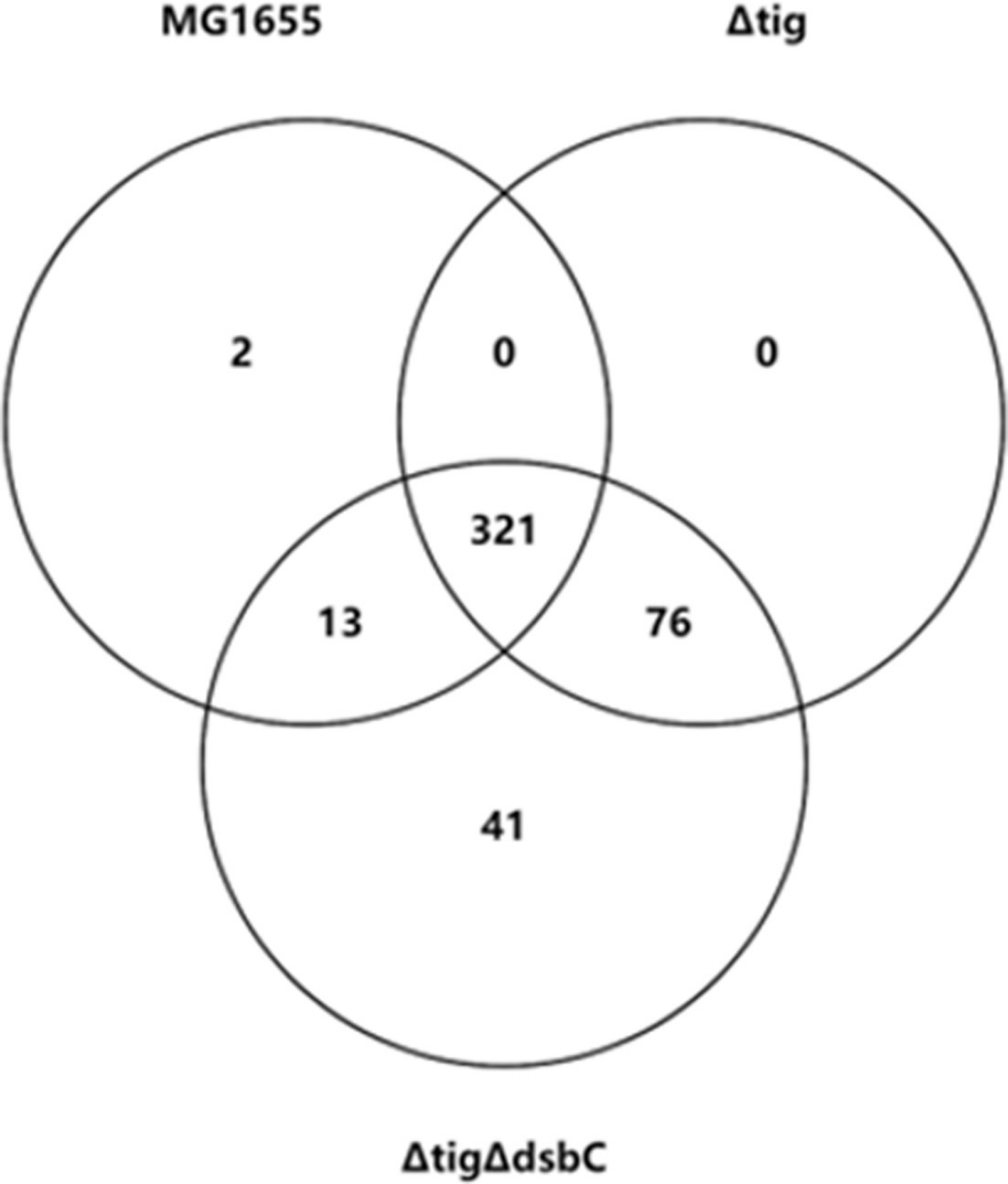
Venn diagram of genes essential for viability in MG1655, MG1655 Δ*tig* and MG1655 Δ*tig* Δ*dsbC*. High-density transposon libraries of MG1655, DRH856 (Δ*tig*) and DRH1292 (Δ*tig* Δ*dsbC*) were sequenced using Illumina, and the essential genes in each strain were predicted using TraDIS. Predicted essential genes were confirmed by examining the insertion sites manually in the Artemis genome browser. The number of essential genes common to different strains is given in the overlapping circles. A list of essential genes in each strain is provided in data S1.

A comparison of the essential genes in each strain revealed that there were 76 genes in MG1655 Δ*tig* and 117 genes in MG1655 Δ*tig* Δ*dsbC* that were not essential in the parental MG1655 strain (Fig. 1 and data S1). All of the 76 additional essential genes in MG1655 Δ*tig* were also essential in MG1655 Δ*tig* Δ*dsbC* but contained transposon insertions in MG1655 Δ*dsbC* (indicating that they were non-essential in this strain). Due to the low number of detected insertions in the MG1655 Δ*dsbC* library, it was not possible to conclude whether any of the remaining 41 essential genes were unique to MG1655 Δ*tig* Δ*dsbC*. However, we did not detect transposon insertions in these genes in the MG1655 Δ*dsbC* library, suggesting that 117 essential genes in MG1655 Δ*tig* Δ*dsbC* are likely the combination of the essential genes in MG1655 Δ*tig* and MG1655 Δ*dsbC*. A handful of genes involved in cell envelope biogenesis or cell envelope stress responses were identified as essential in MG1655 Δ*tig* but non-essential in the parent, including *glmS*, *gmhA*, *lpp* and *mgrB*, and several additional such genes were identified as essential in MG1655 Δ*tig* Δ*dsbC*, including *waaS*, *csgF*, *ariR* and *ymgC*, consistent with the idea that the Δ*tig* and Δ*dsbC* mutations caused a defect in cell envelope biogenesis.

### Isolation of azide-resistant transposon mutants

To isolate azide-resistant transposon mutants, we grew the MG1655 Δ*tig* Δ*dsbC* transposon mutant library on LB plates containing 1.5 mM sodium azide and isolated six azide-resistant colonies. All six of the isolated mutants (which we denoted as *uar* mutants for ‘unlinked azide resistance’) (Table 3) grew on media containing up to 1.5 mM sodium azide and produced a smaller zone of clearing surrounding a filter disc containing 5 µmol of azide compared to the unmutagenized parent. Transduction of the transposon mutations into unmutagenized MG1655 Δ*tig* Δ*dsbC* by selecting for kanamycin resistance resulted in cotransduction of azide resistance, suggesting that transposon insertions caused azide resistance. All six mutations also increased the MIC of azide in strain BW25113 from 0.5 mM to 2–4 mM (Table S1), and the mutations had a similar effect on the azide resistance of parental MG1655 strain, indicating that the mutations did not cause resistance by suppressing the effect of the Δ*tig* or Δ*dsbC* mutations.

**Table 3.** Locations of the transposon insertion sites in azide-resistant mutants

Mutation	Gene*	Location†	Gene product
*uar-1*	*rpsQ* (-)	3448298 (+)	30S ribosomal protein S17
*uar-2*	*rpsQ* (-)	3448296 (-)	30S ribosomal protein S17
*uar-3*	*rpsA* (+)	963307 (+)	30S ribosomal protein S1
*uar-4*	*rpsQ* (-)	3448296 (-)	30S ribosomal protein S17
*uar-5*	*srmB* (+)	2712934 (+)	DEAD-box RNA helicase SrmB
*uar-6*	*nusB* (+)	435524 (+)	Transcription anti-termination factor NusB

* Name of disrupted gene. The ‘+’ symbol indicates that the gene is encoded on the sense strand in the reference genome sequence for *E. coli* K-12 str. MG1655 (NCBI accession NC_000913.3), and the ‘-’ symbol indicates that the gene is encoded on the antisense strand.

† Location of the transposon insertion given as the position in reference sequence NC_000913.3. In the case of the three *rpsQ* mutations, the transposon is inserted downstream of the open reading frame between the termination codon and the Rho-independent transcription terminator. The ‘+’ and ‘-’ symbols indicate that the kanamycin resistance gene is encoded on the sense or antisense strand of the reference sequence.

The *uar* mutations had differing effects on the production and translocation of maltose binding protein (MBP) in the presence of azide (Fig. S2). The *uar-2*, *uar-3, uar-4* and *uar-6* appeared to cause increased steady-state levels of MBP compared to the parent, but production of MBP was undetectable in the *uar-1* and *uar-5* mutants. The presence of azide caused increased accumulation of the precursor form of MBP in the parent, consistent with a defect in SecA-dependent protein translocation. However, precursor MBP could not be detected in the lysates of *uar-2*, *uar-4* and *uar-6* mutants, suggesting that these mutations allow Sec-dependent protein translocation in the presence of azide.

Mapping of the mutations revealed that the *uar-1*, *uar-2* and *uar-4* mutants contained insertions at two distinct sites in the 3′ untranslated region of *rpsQ*, which encodes ribosomal protein S17 (Table 2). The *uar-3* mutant contained an insertion in *rpsA*, which encodes ribosomal protein S1. The *uar-5* mutant contained an insertion in *srmB*, which encodes an RNA helicase involved in rRNA folding, and the *uar-6* mutant contained a mutation in *nusB*, which encodes a transcription anti-termination factor involved in rRNA biogenesis.

### Effect of SecM-induced translational pausing on azide resistance

Production of SecA is regulated post-transcriptionally by the secretion monitor polypeptide SecM, which causes translational pausing [35]. Because all of the *uar* mutations affected translation, we reasoned that they might cause increased azide resistance by affecting SecM-mediated regulation of *secA*. To investigate this possibility, we measured read-through of SecM-induced translational pausing by measuring production of PhoA in cells expressing a SecM-PhoA fusion protein (Fig. S3). The *uar-5* (*srmB*) and *uar-6* (*nusB*) mutations caused decreased PhoA activities compared to the parent strain, suggesting that they caused increased translational stalling at the SecM-arrest sequence. However, the *uar-1*, *uar-3* and *uar-4* (*rpsQ*) and *uar-2* (*rpsA*) mutations caused increased PhoA activity, suggesting that these mutations cause increased read-through of SecM-induced translation arrest. Another explanation was that increased levels of SecM-arrested ribosomes cause azide resistance. However, expression of neither PhoA nor SecM-PhoA from a plasmid significantly affected azide resistance (Fig. S4). Taken together, these results suggested that the *uar* mutations do not cause azide resistance by affecting SecM-induced translational pausing.

### Effect of azide and *uar* mutations on the steady-state level of SecA.

**Fig. 2.**
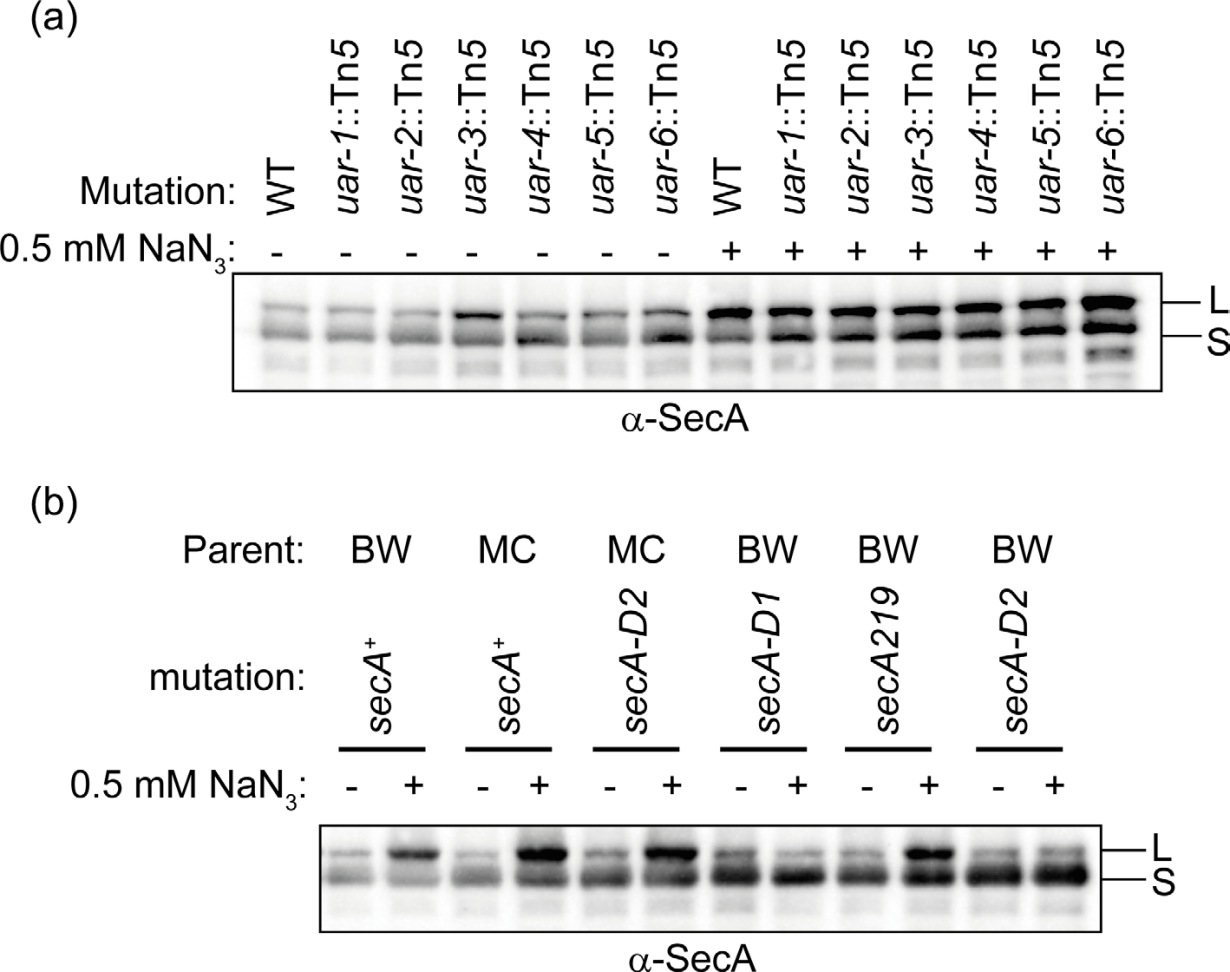
Effect of mutations on the accumulation of the two isoforms of SecA. (**a**) MG1655, CJ107 (*uar-1*), CJ108 (*uar-2*), CJ109 (*uar-3*), CJ110 (*uar-4*), CJ111 (*uar-5*) and CJ112 (*uar-6*) or (**b**) BW25113 (BW), MC4100 (MC), DRH998 (MC4100 *secA-D2*), DRH1116 (BW25113 *secA-D1*), DRH1122 (BW25113 *secA219*) and DRH1127 (BW25113 *secA-D2*) were grown in LB in the absence (−) or presence (+) of 0.5 mM sodium azide to mid-log phase. Equal amounts of cell lysates were resolved by SDS-PAGE and analysed by Western blotting using antiserum directed against SecA. The running positions of the large (L) and small (S) isoforms of SecA are indicated.

Although the *uar* mutations did not appear to affect SecM-mediated regulation of *secA*, it was nonetheless possible that these mutations caused changes in the steady-state levels of SecA. To investigate this possibility, we examined the effect of azide and the *uar* mutations on the steady-state levels of SecA by Western blotting. Consistent with previous reports [26], SecA migrated as two distinct isoforms with different apparent molecular weights in SDS-PAGE (Fig. 2). In the absence of azide, there was no detectable difference in the steady-state levels of SecA or the relative abundance of the two isoforms between the parent strain and the isogenic *uar* mutants. However, growth in the presence of 0.5 mM sodium azide caused a substantial accumulation of the large SecA isoform (Fig. 2a). Furthermore, all of the *uar* mutations caused an increase in the relative abundance of the smaller SecA isoform compared to the isogenic parent strain.

### Effect of mutations in the *secA* gene that confer azide resistance on the production of the small SecA isoform

We speculated that the *uar* mutations could cause azide resistance by a similar mechanism to *secA* mutations that confer azide resistance (i.e. *azi* mutations), and if so, the *secA* mutations might have a similar effect on the relative levels of the two SecA isoforms. To investigate this possibility, we determined the steady-state levels of the SecA isoforms in several azide-resistant *secA* mutants by Western blotting. Two of the *secA* alleles (*secA-D1* and *secA-D2*) were isolated *de novo* for this study by selecting for spontaneous mutants of *E. coli* MC4100 on plates containing 3 mM sodium azide. All of the *secA* mutations caused an increased proportion of the small isoform in two different parental lines of *E. coli* (BW25113 and MC4100) in the presence of 0.5 mM sodium azide (Fig. 2b). Furthermore, the relative levels of the small SecA isoform were more pronounced in the *secA* mutants than in the *uar* mutants, consistent with the higher level of azide resistance conferred by these mutations. These results suggest that the *azi* and *uar* mutations confer azide resistance by similar mechanisms.

### Identification of the SecA isoforms

**Fig. 3.**
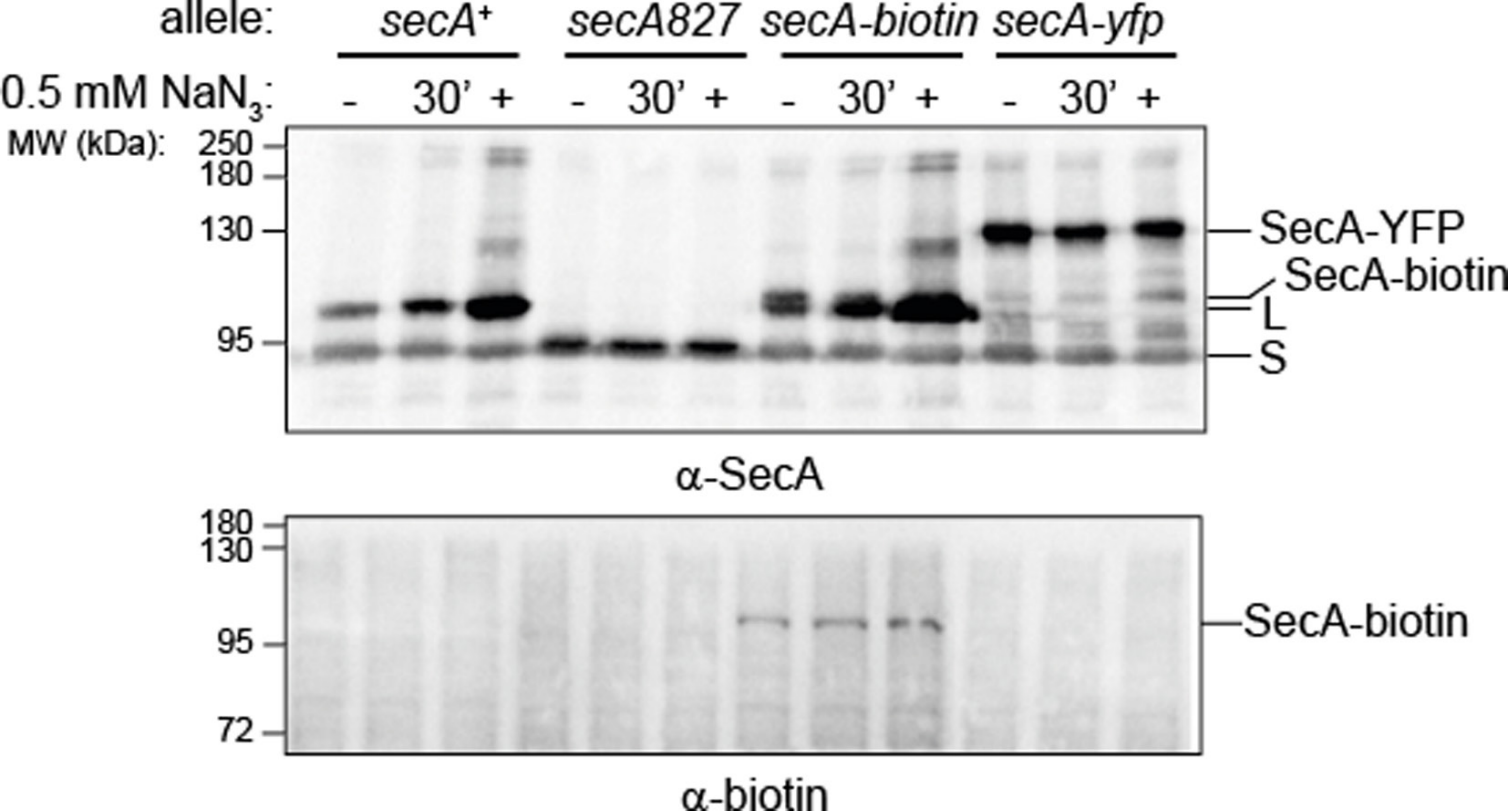
Identification of the small isoform of SecA. MC4100 (*secA^+^*), DRH1064 (*secA827*), DRH839 (*secA-biotin*) and DRH991 (*secA-yfp*) were grown in LB to mid-log phase in the absence or presence of 0.5 mM sodium azide. Cultures grown in the absence of azide were split at OD_600_ 0.5 and treated with 0.5 mM sodium azide for 30 min before harvesting. Equal amounts of cell lysates were resolved by SDS-PAGE and analysed by Western blotting using anti-SecA antiserum (upper panel) or using HRP-coupled streptavidin (lower panel). The positions of the large (L) and small (S) isoforms of wild-type SecA, full-length SecA-biotin (SecA-biotin) and full-length SecA-YFP (SecA-YFP) are indicated. The expression of SecA in strains DRH839 and DRH991 was induced using 1 mM IPTG.

A previous study by our group suggested that insertion mutations that cause the removal of the CTT of SecA reduce the susceptibility of *E. coli* to sodium azide [9]. We therefore reasoned that the smaller isoform could be a truncated form of SecA. To investigate this possibility, we compared the sizes of the two SecA isoforms in cells producing different variants of SecA, including (i) full-length SecA, (ii) a truncated SecA protein lacking the C-terminal 74 amino acids (SecA827) [36], (iii) a SecA protein containing a C-terminal fusion to a 15 amino acid biotin-attachment peptide (SecA-biotin) [37] and (iv) a SecA protein containing a C-terminal fusion to the yellow fluorescent protein (SecA-YFP). Cells producing SecA827 produced a single band that cross-reacted with SecA-specific antibodies and migrated with an apparent mass similar to the smaller SecA isoform (Fig. 3a), indicating that the larger band was full-length SecA and that the CTT was required for production of the two isoforms. Cells producing SecA-biotin as the sole version of SecA produced three bands that cross-reacted with SecA antibodies: two bands identical in size to the large and small SecA isoforms and a slightly larger band, but only one of these bands cross-reacted with HRP-conjugated streptavidin (Fig. 3b), suggesting that the smaller isoforms lacked the C-terminal biotin. (If the smaller isoform was the result of an N-terminal truncation or a different type of posttranslational modification, two bands should cross-react with streptavidin.) Finally, cells producing SecA-YFP produced only two prominent SecA isoforms: a large isoform consistent with the size of the full-length SecA-YFP fusion protein and a small isoform consistent with the size of SecA827 (Fig. 3a). These results suggested that the large isoform is full-length SecA, while the small isoform lacks the CTT. The MIC of cells expressing SecA827 as the sole copy of SecA was moderately higher than that of the parent (1 mM compared to 0.5 mM) (Table S1), as was the MIC of cells coexpressing both full-length SecA and a truncated SecA lacking the C-terminal 69 amino acids (SecAΔCTT). However, the MIC of cells overexpressing full-length SecA from an IPTG-inducible promoter was identical to the parent (Table S1). Taken together, these results suggest that the increased azide resistance of the mutants could be explained, at least in part, by the increased production of the small SecA isoform.

## Discussion

We have identified the first mutations, to our knowledge, that confer resistance to sodium azide in *E. coli* that are not located in the *secA* gene. All of the mutations are in genes that affect ribosome biogenesis, and most of the mutations significantly improve translocation of MBP in the presence of sodium azide, suggesting a shared mechanism of resistance between the *uar* mutations. In addition, all of the *uar* mutations cause increased production of the small isoform of SecA when cells are grown in the presence of sodium azide, and mutations in *secA* that confer azide resistance also caused increased accumulation of the small SecA isoform, suggesting a shared mechanism between the *uar* and *secA* mutations. The small SecA isoform consists of a C-terminally truncated SecA protein that lacks the CTT, and previous studies indicate that removal of the CTT affects multiple activities of SecA. For example, the basal ATPase activity of the truncated form of SecA is several-fold higher than that of full-length SecA [6], and the CTT is required for stable homodimerization of SecA [38,39]. Previous work by our group indicates that azide disrupts the structure of the C-terminal MBD at the extreme C-terminus and that transposon mutations removing the CTT confer a competitive advantage during growth in the presence of azide [9], consistent with the results presented in this study.

However, the level of resistance displayed by the *uar* mutants cannot be fully explained by the increased production of the small SecA isoform. For example, most of the *uar* mutants caused an increase in the MIC of azide of up to 4 mM – equal to that of many previously characterized *secA* mutants – while increased production of C-terminally truncated SecA causes only a moderate increase in the MIC. Furthermore, none of the *uar* mutations affect genes encoding components of the Sec machinery even though many affect translocation of MBP in the presence of azide, raising the possibility that azide inhibits translocation indirectly *in vivo*. For example, it is possible that inhibition of Sec-dependent translocation could be caused by a stress response resulting from inhibition of respiration. Indeed, inhibition of respiration by azide in *Saccharomyces cerevisiae* induces a stress response that strongly inhibits protein synthesis [40,41], raising the possibility that azide could indirectly inhibit protein biogenesis in other organisms such as *E. coli*.

Our results do not reveal the source of the small SecA isoform. One possibility is that the smaller isoform is the result of proteolytic processing. Consistent with this possibility, mutations disrupting proteases, such as the oligopeptidase PrlC and the AAA+ Lon protease, can suppress many Sec defects *in vivo* [42,43], and at least one other component of the Sec machinery, FtsY (which encodes the SRP-receptor protein), undergoes proteolytic processing [44]. However, it is not clear why the *uar* mutations, all of which affect protein synthesis, could cause increased posttranslational cleavage of full-length SecA. Another possibility is that the small SecA isoform could be an alternative translation product. Indeed, several genes in *E. coli* make two products as a result of programmed translational frameshifting, including *copA* [CopA and CopA(Z)] and *dnaX* (the tau and gamma subunits of the DNA polymerase III holoenzyme) [45,48]. Production of the small SecA isoform by programmed translational frameshifting might also explain why such a large number of mutations in genes affecting protein synthesis can suppress defects in protein translocation, including mutations in *rpsA*, *nusB*, *rpmH*, *rpsO*, *infB* and *suhB* [49,53] and why subinhibitory concentrations of chloramphenicol can suppress some Sec defects [54].

## Supplementary material

10.1099/mic.0.001707 Supplementary Material 1.

10.1099/mic.0.001707 Supplementary Material 2.

## Abbreviations

CTT: C-terminal tail
FLD: flexible linker domain
MBD: metal-binding domain
MBP: maltose binding protein
SPRI: solid-phase reversible immobilization
TraDIS: transposon-directed insertion-site sequencing
