# Supplementary material for: Mutations that confer resistance to sodium azide affect the levels of two physiological isoforms of SecA in Escherichia coli
Source: Microbiology (Reading). 2026 May 15;172(5):001707. doi: 10.1099/mic.0.001707 (PMC13178939; doi:10.1099/mic.0.001707)
Supplement: Supplementary Material 2. [file mic-172-01707-s002.pdf]

SUPPLEMENT.

Supplemental table S1. Minimum inhibitory concentrations (MIC) of sodium azide for *E. coli* strains.

Background strain	Relevant genotype	IPTG[a]	MIC (mM)[b]
BW25113	WT	-	0.5
BW25113	WT	10 µM	0.5
CJ113	BW25113 *uar-1*	-	4
CJ114	BW25113 *uar-2*	-	4
CJ115	BW25113 *uar-3*	-	2
CJ116	BW25113 *uar-4*	-	4
CJ117	BW25113 *uar-5*	-	4
CJ118	BW25113 *uar-6*	-	4
DRH1116	BW25113 *secAD1*	-	4
DRH1122	BW25113 *secA219*	-	>4
DRH1127	BW25113 *secAD2*	-	4
DRH1070	BW25113 $secA^{+}$ p_{trc}-*secA832*	10 µM	1
MC4100	WT	-	0.5
DRH997	MC4100 *secAD1*	-	>4
DRH1075	MC4100 *secA827::*IS*1*	-	1

DRH745	MC4100 Δ*secA* p_{trc}-*secA* (IPTG-inducible)	1 mM	0.5

[a]MIC values were determined using plates containing IPTG at the indicated concentration.

[b]MIC values for sodium azide were determined by streaking strains on LB plates containing IPTG at concentrations ranging from 0.25-4 mM. The MIC was taken to be the lowest concentration that prevented formation of individual bacterial colonies.

Supplemental figure S1.

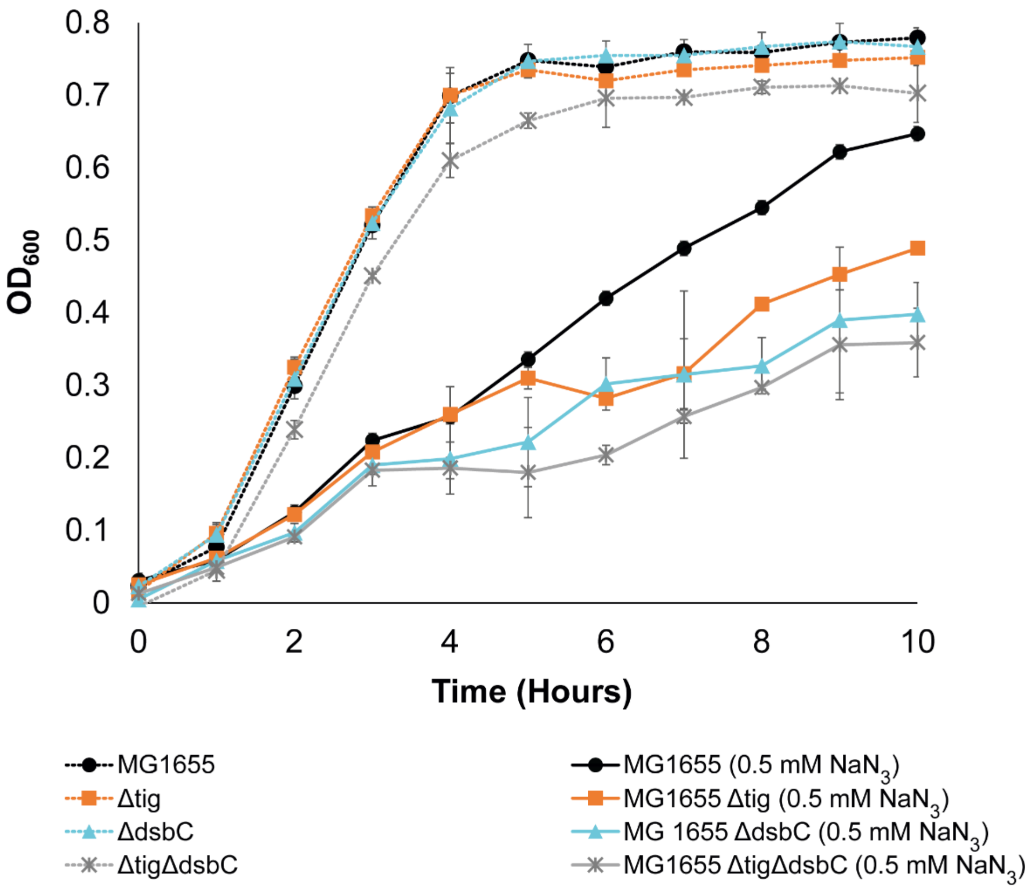

Supplemental figure S1. Effect of Δ*tig* and Δ*dsbC* mutations on susceptibility of *E. coli* to sodium azide. Growth curve of MG1655 (black circles), MG1655 Δ*tig* (orange squares). MG1655 Δ*dsbC* (light blue triangles), and MG1655 Δ*tig* Δ*dsbC* (gray Xs) in LB (dotted lines) and LB containing 0.5 mM sodium azide (solid lines). OD_{600} was measured hourly. The average of three independent cultures is plotted. Confidence intervals represent one standard deviation.

Supplemental figure S2.

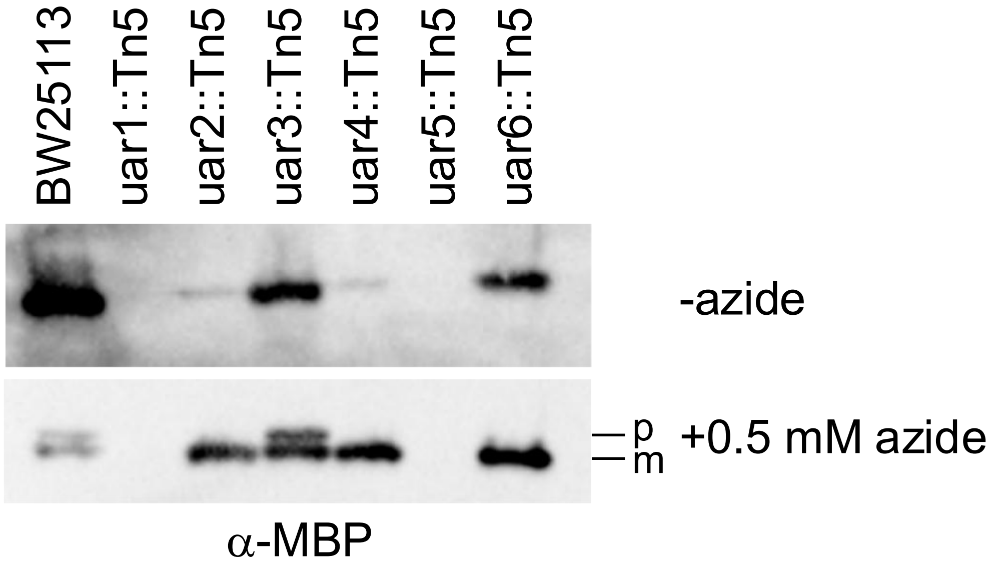

Supplemental figure S2. Effect of *uar* mutations on translocation of MBP. *E. coli* strains BW25113, CJ113 (*uar-1*::Tn*5*), CJ114 (*uar-2*::Tn*5*), CJ115 (*uar-3*::Tn*5*), CJ116 (*uar-4*::Tn*5*), CJ117 (*uar*-5::Tn*5*) and CJ118 (*uar-6*::Tn*5*) were grown in LB containing 0.2% maltose to mid-log phase in the absence or presence of 0.5 mM sodium azide. Lysates of the cells were adjusted to OD_{600} 10, resolved by SDS-PAGE and probed by western blotting against maltose binding protein (MBP). The running positions of precursor (p) and mature length (m) MBP are indicated in the azide-treated sample.

Supplemental figure S3

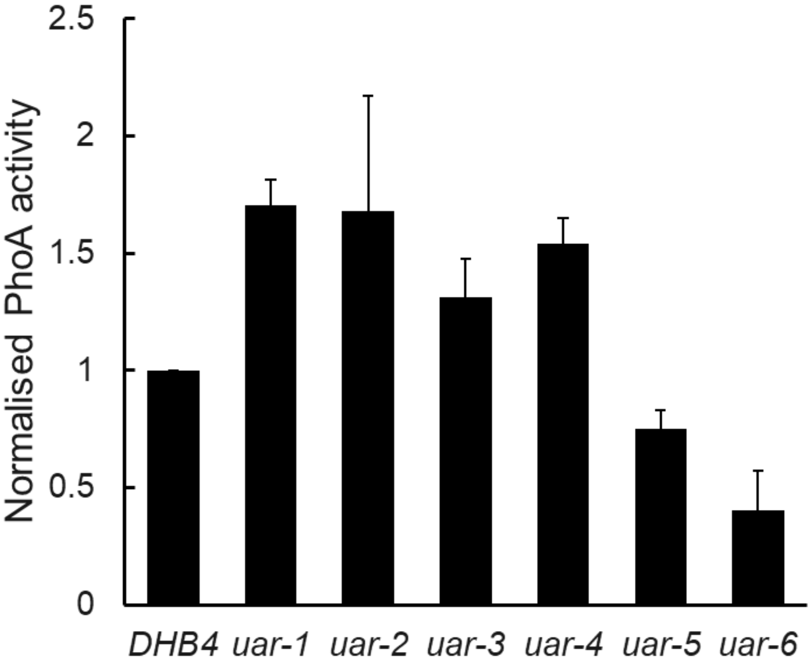

Supplemental figure S3. Alkaline phosphatase activity of the azide-resistant mutants. DHB4 or DHB4 containing the indicated *uar* mutation was transformed with plasmid pDH736, which produces a SecM-PhoA fusion protein. The strains were grown in LB in the presence of in the presence of 1 mM IPTG and 0.5 mM sodium azide at 37°C, and read-through of the SecM-induced translational arrest was determined by measuring alkaline phosphatase (PhoA) activity. The activities represent the normalised average of three biological replicates measured on three separate days. Error bar represent one standard deviation.

Supplemental figure S4.

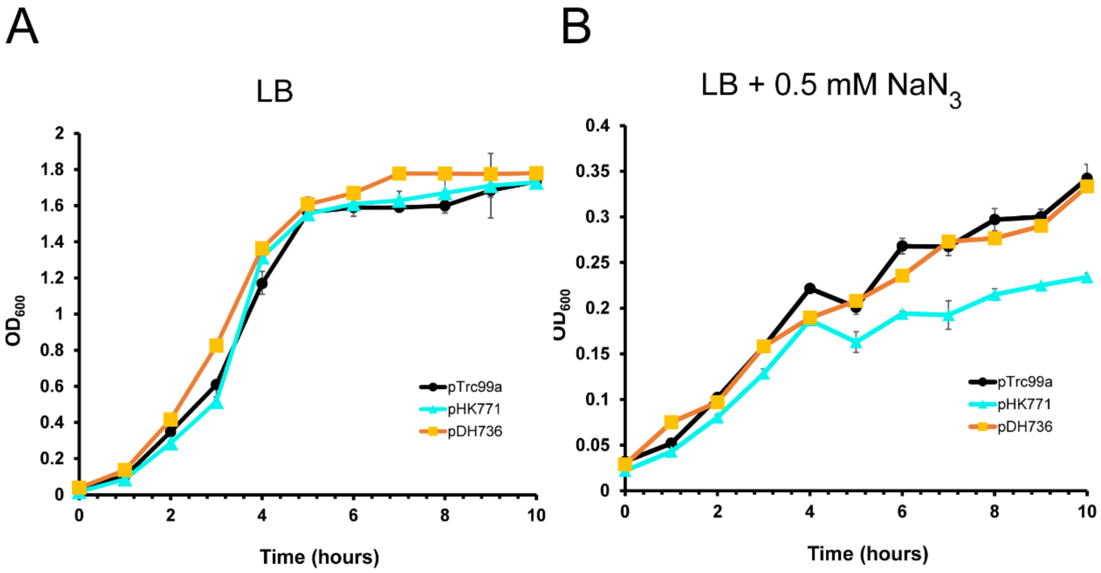

Supplemental figure S4. Expression of SecM does not affect the azide resistance. MC4100 containing plasmid pTrc99a (black circles), pHK771 (cyan triangles) or pDH736 (orange squares) was grown in LB in the absence (A) or presence (B) of 0.5 mM sodium azide in LB, and the OD_{600} of the culture was measured hourly. Confidence intervals represent the standard deviation of 3 independent experiments.
